# Medical Gossip

**Published:** 1862-07

**Authors:** 


### Art. X.—MEDICAL GOSSIP.

A nucleus for a Female Medical College has been formed in London, and an appeal will shortly be made to the public and the profession for help.

Were the General Council of Medical Education in the habit of unmuzzling their wisdom, we should look forward with no little interest to the time when this subject will probably have to be discussed by them. The Council, however, still persists in its injurious reticence. Nothing but wisdom in its most concentrated and least easily digestible form is to be doled out by them. As might have been foreseen, very little time has elapsed before the confidence of the profession, however unwillingly, has been shaken to the foundation, not only in the judgment of the Council, but also, which is infinitely more serious, as to its decisions being at all times governed by a strict regard to duty. The glaring conduct of the Council of the Royal College of Surgeons in issuing a series of regulations differing in several important respects from the principles of education and recommendations laid down by the General Council, was early made the subject of discussion in its recent meeting. But although the regulations referred to adopt a *minimum* of education less than that recommended by the General Council, and specify times from which to date the commencement of that education different from, and rendering nugatory the important results sought to be obtained from the period fixed by them, the Council, by a majority, which included the President of the College of Surgeons, Mr. Lawrence, one of the Council of the College, Mr. Arnott, another of the Council, and one of the Court of Examiners, refused to interfere. There may be good grounds for such a course; but by the exclusion of reporters from the meetings of the General Council, when discussing questions which are of moment to the profession, not merely from the results, but also from the method and for the reasons by which those results are arrived at, the uninitiated can only see an important principle advocated by the General Council, and approved by the profession, deliberately set aside by an examining and licensing body, and this undoubted dereliction of duty towards the profession suffered to pass without protest. The profession also cannot fail to notice that among the majority thus stultifying the recommendations of the General Council are the names of three of its members who had already, it is to be presumed, committed themselves to an approval of, and were in part responsible for, the regulations of the College of Surgeons objected to.

The *British Pharmacopœia* it appears is now ready for publication, but certain legal difficulties in the way of copyright and the promulgation of its authority, have still to be got rid of before the work can be given to the world. 1600*l.* have been disbursed by the General Council for the preparatory expenses of the Pharmacopœia. It is to be hoped that the printing and publishing arrangements of the work will be somewhat more satisfactorily carried out than in the case of the *Register.* The sale of the latter unfortunate work amounted to 768 copies in 1860, but only to 532 in 1861.

The income of the General Council, in 1860, was 4134*l.* 5s. 7d.; its expenditure 5461*l.* 9s. 9d. In 1861 the income was 4871*l.* 11s. 4d., and the expenditure 4749*l.* 6s. 11d. For the present year it is estimated that the income will be 4480*l.* and the expenditure 4366*l.*

The Medical Acts Amendment Committee, appointed by the General Council, recommend the following amendment to Clause 40:—"Any person who shall take or use the name or title of Physician, Doctor of Medicine, Licentiate in Medicine and Surgery, Bachelor of Medicine, Surgeon, General Practitioner, or Apothecary, or any name, title, addition, or description implying that he is qualified to practise any branch of Medicine or Surgery, *such name, title, addition, or description not having been granted by any of the licensing bodies mentioned in Schedule A of this Act*, shall, upon summary conviction for any such offence, pay a sum not exceeding 20*l*." It is added, "The heading of Section 40, so altered, might be—Penalty for using Medical or Surgical designations by persons not qualified under Schedule A." The Committee appointed to examine how far certificates granted to students for attendance on hospitals or lectures are granted on *bonâ fide* attendance, report that:—

"In the Universities of Cambridge and Dublin, in which the great majority of the medical students are passing their terms in Arts, regularity in attendance is ensured; in Dublin, by roll-call by such professors of the School of Physic over whom the University has direct jurisdiction; and in Cambridge by inscription of names, and by delivery of cards bearing the student's name.

"In Oxford the majority of the examiners decide on the question as to how far the certificate is satisfactory.

"The remaining bodies may be divided into two categories. In one, various modes are adopted to ensure regularity of attendance, which must, to a great degree, effect the object. Under this head may be placed the Universities of Edinburgh, Aberdeen, Glasgow, and Durham, and the Royal College of Surgeons of Edinburgh.

"In the second category may be placed those bodies which rely on the care and trustworthiness of those teachers whose names appear on the certificate. Under this head may be enumerated the Universities of London and St. Andrews; the Royal College of Physicians, London; the Royal College of Surgeons of England; the Faculty of Physicians and Surgeons of Glasgow; the Society of Apothecaries of London, and the Apothecaries' Hall, Dublin.

"The Committee are not yet in a position to state what is the practice, in reference to this question, of the King and Queen's College of Physicians of Ireland, the Royal College of Surgeons of Ireland, and the Queen's University in Ireland. It may, however, be stated, that in those bodies to which Schools of Medicine are attached, attention is paid—though by the adoption of different methods—to ensure a *bonâ fide* attendance of those students to whom certificates are granted. Great difficulty arises on the part of those bodies by which licences are granted, yet where no School exists under their immediate jurisdiction, in determining the question of *bonâ fide* attendance. It is plain, further, that in cases of attendance at private schools, the certificates of the teachers who grant them have to be received by the licensing body on the responsibility of the grantors of the document. It is not to be lost sight of, that licensing bodies have every day presented to them certificates from schools at a distance from them, and over which they have no power. These considerations, especially when the present and obvious overloading of the curriculum of study enforced in these countries is borne in mind, make the attempt at an effective supervision of class-room or hospital attendance a matter of great difficulty. In the opinion of the Committee, the means most likely to lessen the evils which necessarily flow from a loose system of granting or receiving certificates, must be sought for in some important modifications on the part of the licensing bodies of the curricula now enforced."

The returns made to the General Council of the number of candidates examined by the different licensing bodies and Universities, although still imperfect, show that 1609 were examined in 1861, of whom 200 were rejected.

The difficult questions arising out of the removal of St. Thomas's Hospital are still unsettled, and the Governors have been subjected to no small amount of public odium, for the eccentric and unsatisfactory way in which they are proceeding, and have proceeded, with the grave duties entrusted to them. As matters stand, unless impediments arise in the course of the Bill now pending in Committee, authorizing the Governors to effect, or perfect sale, the present hospital must be vacated on or before the 26th of this month. It would appear that negotiations opened with the Charing Cross Railway Company, the compulsory purchasers of the site, to arrange terms whereby the patients might remain till a new hospital could be built (subject to the occupation of any part of the land required by the railway company) have entirely failed. The company first demanded a rent of 18,000*l*. for one year, and that the hospital should pay all costs of arbitration, &c., these costs the railway company being liable to pay, either under the Land Clauses Act, or by the decree of Vice-Chancellor Wood. The reply of the Governors to this proposition was to the effect, that they could not interfere with the question of costs, which must take the course determined by law, but they would give a rent of 12,000*l*. per annum for two years, subject to a six months' notice after the first year; this 12,000*l*. being the interest of the total purchase-money for the whole hospital at the usual rate of 4 per cent. This was refused; but a further proposition, at the request of the hospital, was made by the railway company. This was a rent of 16,000*l*. for one year, coupled, at the same time, with the payment of costs under certain conditions, amounting to about 10,000*l*. more, the hospital to pay also the costs of the company in the appeal to the Lord Chancellor. The grand committee of the hospital agreed to give the 16,000*l*. per annum, but declined to interfere with the costs in any manner whatever. The negotiation then terminated, and the Governors are now attempting to provide temporary accommodation for the patients in other hospitals (under the care of their own physicians, surgeons, and nurses), and elsewhere. Whatever space may be granted in existing hospitals can only be given by the exclusion of other cases having a more legitimate claim for admission there. Among the arrangements, if not the only arrangement, about to be made elsewhere, is the provision of 200 beds in the Surrey Music Hall. Upon this part of the scheme of the Governors the *Times* (June 18) has justly remarked, after pointing out that it implies the loss to the sick public of no less than 300 beds—the accommodation in St. Thomas's being for 500 patients:—

"This consideration alone should make the House of Lords pause before adopting the Bill in its present form. The loss of so large a number of beds is no trifle, and will be severely felt by those classes who look to the nearest hospital as their only asylum in the event of accident or disease. But it is not merely space that is sacrificed. It is quite impossible that an edifice specially designed for concerts can be adapted, without long delay and ruinous expense, to the proper reception of the sick and wounded. Such a conversion cannot be otherwise than a make-shift. It will involve a great diminution of comfort to patients, and the purely gratuitous inconvenience of two moves instead of one. What effect it may have in breaking up the medical school attached to the present establishment we cannot say, but it will certainly reduce their opportunities for clinical study in the proportion of 2 to 5. Of course, if all these evils were unavoidable, they must be borne as best they might; but this is far from being the case. The railway company never demanded more than a small part of the ground in question. They would have been too glad to be without the hospital itself, and took it only under compulsion. All the land they wanted they have already occupied for weeks, and if the contract for the remainder could even now be broken off upon fair terms, they would probably consider themselves gainers. There cannot, therefore, be any urgent necessity for completing the conveyance. All parties can well afford to wait till a new and permanent edifice is ready to receive all the inmates of the old one. If the intervention of Parliament is essential, Parliament can fix its own conditions, and ought to insist upon some guarantee for a final choice that will be satisfactory to the public. It might be sufficient for this purpose to make the selection of a future site subject to approval by the Charity Commissioners, after full public notice given of the competing plans, or there may be other ways of securing the same thing. What is really important is, that such a question should not be practically settled by secret committees, or made subservient to the convenience of a few individuals, but discussed with paramount reference to the great objects of this princely charity."

A curious and most amusing case, and one of considerable interest to the profession, was heard in the Court of Queen's Bench, Westminster, before Lord Chief-Justice Cockburn, and

Justices Crompton, Blackburn, and Mellor, on the 9th of May last:—

Mr. Coleridge, Q.C., moved for a rule calling upon T. C. Savill and G. Coker, the printer and publisher of the *Lancet*, to show cause why a criminal information should not be filed against them for a libel published in the *Lancet* on the 22nd of March last in the form of a review of a book published by the applicant. Dr. Hastings, in his affidavit, stated that he had been for about twenty years a graduate of Edinburgh, and during his career as a physician in this metropolis he had devoted himself to enlarging the bounds of the pharmacopœia, and in the year 1843 he published a book, in which he showed that pulmonary consumption might be successfully treated with naphtha. He had since published another book, in which he showed that oxalic acid, fluoric acid, and phosphorus might be successfully employed for the same purpose, and these remedies had been tried by persons of the highest distinction in the medical world. Among others who had consulted Dr. Hastings the learned counsel mentioned Mr. Guthrie, Dr. Latham, Mr. Salmon, and Mr. Lawrence, the eminent surgeons; and Mr. Tuffnell, the well-known Inspector of Schools, was cited as having spoken in high terms of Dr. Hastings' discoveries. In the year 1861 Dr. Hastings discovered that the *excreta* of reptiles possessed very powerful properties, but he had no intention of making his experiments public. It happened, however, that a patient attacked him before the College of Physicians for giving him colourless water, and the College of Physicians having heard the complaint, with the Solicitor-General as their assessor, dismissed the complaint without calling upon Dr. Hastings for an answer. On that occasion Dr. Hastings told the College of Physicians what he had given, and having thus made it known, he, in the present year, published a book under the title of "An Inquiry into the Medicinal Value of the Excreta of Reptiles in Phthisis and some other Diseases." On the 22nd of March last the book in question was reviewed in the *Lancet*, and the learned counsel contended that the review far exceeded the bounds of fair criticism, and showed that the writer was actuated by personal hostility to the author. The learned counsel then read the review at length. It was written in a somewhat sarcastic style, and began by referring to Dr. Hastings' first work, published nearly twenty years ago, entitled "Pulmonary Consumption successfully Treated with Naphtha," and which purported to be by John Hastings, M.D., Senior Physician to the Blenheim-street Free Dispensary. An extract from the work was given, in which naphtha was described as "little less than a specific in the earlier stages of the disease," and the author expressed himself as "most sanguine that even in the latter stages of the disease a restoration to health may generally be calculated upon." The review stated that the result of the investigation was that "the vaunted specific was utterly destitute of the virtues ascribed to it. At any rate, the fact was, in ten years' time the public became used up, *quoad* naphtha. Either the latter ceased to cure, or people refused to be cured by it." The article went on to say that "such consequences were, of course, serious. In 1854, therefore, the profession were treated to a new work by Dr. Hastings on the cure of phthsis." The review then stated that "the author confessed that the 'treatment of consumption by naphtha or pyroxylic acid spirit, which was attended with much success,' was so no longer;" and an extract was given from Dr. Hastings' new work, in which he said "that for some time he regarded it (naphtha) almost as a specific in phthisis;" but "my subsequent success has not equalled that which attended the first year of its trial." The review went on to say, "Naphtha, then, being no longer equal to its duty, Dr. Hastings introduced to the public, in his new treatise, fluoric and oxalic acids as the agents upon which it might safely rely in the treatment of pulmonary *tuberculosis*." Extracts were then given from Dr. Hastings' new work, in which he spoke very highly of the effects of his new agents, which he said produced the "most satisfactory results." The review went on to say, that "by the time this announcement was made, the profession generally had become accurately acquainted with the measure of Dr. John Hastings;" but "whether the public was equally deaf to the charmer is another matter. However, it must have become impatient at last, for a new specific has clearly been demanded. Nor has the great discoverer been inexorable. He has once more enriched the literature of medicine, and this by a treatise which, in a certain sense, may be described as of no common character." The reviewer went on to ask, "With our previous knowledge of the learned doctor, can we feel surprised at the announcement that he has at length 'found in the *excreta* of reptiles agents of great medicinal value in numerous diseases where much help was needed?'" The reviewer then ridiculed the applicant's alleged discoveries, and quoted an extract, in which Dr. Hastings wrote, "My earliest trials were made with the *excreta* of the boa constrictor, which I employed, in the first instance, dissolved simply in water. A gallon of water will not dissolve two grains, and yet, strange as the statement may appear, half a teaspoonful of this solution rubbed over the chest of a consumptive patient will give instantaneous relief to his breathing." The review concluded thus:—"But, like all great men, Dr. Hastings has had his persecutors ; one of so pertinacious a character as to have compelled the College of Physicians, within the last six months, to summon Dr. Hastings before the Censors' Board to answer the complaint of acting in a way derogatory to his profession. This is no secret, for in the preface of the work the author himself alludes to it. But we must have done with this disreputable and annoying topic. What can the public be thinking about, we would ask, when it supports and patronizes such absurd doings ? Will there still continue to be found persons ready to allow their sick friends to be washed with a lotion of serpent's dung ? It may be so. Naphtha, oxalic acid, and this precious excrement are the expression of that eternal folly which must be fed.

"Populus vult decipi et decipiatur."

Lord Chief Justice Cockburn, during the reading of the review complained of, observed that the writer might have honestly believed that the applicant's alleged discoveries were all moonshine. He might honestly have thought Dr. Hastings was over-sanguine or over-credulous in supposing that the excrement of reptiles could produce such results ; and that those who took a more sober view of things might say it was all moonshine. Mr. Coleridge contended that the effect of the article was to charge the applicant with being a quack. Lord Chief Justice Cockburn said that great service was done to the public by denouncing quackery, provided it were done in a fair spirit.

Mr. Coleridge said there was nothing like oil for sharpening a razor, and the most offensive things might be said with a bland, oily face by those who knew how. The learned counsel complained particularly of the allusion to the inquiry which had taken place before the College of Physicians, and the omission to mention that the charge had been dismissed without calling upon Dr. Hastings for an answer—a fact which must have been well known to the writer of the review.

Lord Chief Justice Cockburn said he thought there were some expressions which might have been omitted ; but with respect to the concluding paragraph of the review (upon which the learned counsel seemed to lay some stress), his Lordship thought that was rather a libel upon the public.

Mr. Coleridge submitted that the article imputed dishonest motives.

Lord Chief Justice Cockburn said, Dr. Hastings has brought forward his three remedies one after another, and, when he had himself given up the first two, he brought forward a third—the excreta of reptiles. It might be that he had discovered a remedy, and, if so, truth would prevail in the end ; but it was not to be wondered at that the matter was treated rather sarcastically when the public were told that phthisis could be cured by the dung of snakes. The article might be a libel, but the applicant had the ordinary remedy, and the Court ought not to interfere, except where there was *mala fides*, or some sinister motive.

The other Judges concurred.—Rule refused.

Death has been busy in the profession during the past quarter. The loss of Mr. Stanley, Dr. J. O. McWilliam, C.B., and Mr. Wakley leaves a great hiatus among the ranks of our best known and most admired leaders. Mr. Stanley was struck down with apoplexy while going his round in the wards of St. Bartholomew's, in the afternoon of the 24th of May, and death occurred within an hour of the attack. He died at the post he had so long and ably filled. Mr. Stanley was Surgeon Extraordinary to the Queen and Senior Surgeon of his hospital. He was also the author of a treatise "On Diseases of the Bones," a work on "The Mode of Performing the Lateral Operation of Lithotomy," and a "Manual of Practical Anatomy," and several scattered papers. We trust in our next number to present our readers with memoirs of Dr. McWilliam and Mr. Wakley. Mr. Wakley's life is that of the social and political history of the profession for half-a-century.

---

